# Identification of Electronic and Structural Descriptors of Adenosine Analogues Related to Inhibition of Leishmanial Glyceraldehyde-3-Phosphate Dehydrogenase

**DOI:** 10.3390/molecules18055032

**Published:** 2013-04-29

**Authors:** Norka B. H. Lozano, Rafael F. Oliveira, Karen C. Weber, Kathia M. Honorio, Rafael V. Guido, Adriano D. Andricopulo, Albérico B. F. Da Silva

**Affiliations:** 1 Instituto de Química de São Carlos, Universidade de São Paulo, São Carlos, SP 13566-590, Brazil; E-Mail: nbhl_@hotmail.com; 2 Departamento de Química, Universidade Federal da Paraiba, João Pessoa, PB 13083-970, Brazil; E-Mails: rfarias.quimica@gmail.com (R.F.O.); karen@quimica.ufpb.br (K.W.C.); 3 Centro de Ciência Naturais e Humanas, Universidade Federal do ABC, Santo Andre, SP 09210-170, Brazil; E-Mail: kmhonorio@usp.br; 4 Escola de Artes, Ciências e Humanidades, Universidade de São Paulo, São Paulo, SP 03828-000, Brazil; E-Mail: kmhonorio@usp.br; 5 Instituto de Física de São Carlos, Universidade de São Paulo, São Carlos, SP 13560-590, Brazil; E-Mails: rvcguido@yahoo.com (R.V.G.); aandrico@ifsc.usp.br (A.D.A.)

**Keywords:** adenosine compounds, antileishmanial activity, glyceraldehyde 3-phosphate dehydrogenase, DFT, multivariate regression

## Abstract

Quantitative structure–activity relationship (QSAR) studies were performed in order to identify molecular features responsible for the antileishmanial activity of 61 adenosine analogues acting as inhibitors of the enzyme glyceraldehyde 3-phosphate dehydrogenase of *Leishmania mexicana* (*Lm*GAPDH). Density functional theory (DFT) was employed to calculate quantum-chemical descriptors, while several structural descriptors were generated with Dragon 5.4. Variable selection was undertaken with the ordered predictor selection (OPS) algorithm, which provided a set with the most relevant descriptors to perform PLS, PCR and MLR regressions. Reliable and predictive models were obtained, as attested by their high correlation coefficients, as well as the agreement between predicted and experimental values for an external test set. Additional validation procedures were carried out, demonstrating that robust models were developed, providing helpful tools for the optimization of the antileishmanial activity of adenosine compounds.

## 1. Introduction

Leishmaniases are diseases caused by the intracellular protozoan parasite *Leishmania*. There are an estimated 1.5–2 million new cases per year, of which up to 500,000 are visceral leishmaniasis (VL), the fatal version of the disease. Left untreated, it causes a global annual mortality estimated at 59,000 [1]. According to disease burden estimates, leishmaniasis ranks third in disease burden in disability-adjusted life years caused by neglected tropical diseases and is the second cause of parasite-related deaths after malaria [2]. For a variety of reasons, it is not receiving the deserved attention given its high occurrence [3].

The first-line treatments for VL since the 1930s are the pentavalent antimonials, although these compounds are toxic and resistance has been an increasing problem in India [4]. While significant progress has been made in the last 10 years, with the approval of amphotericin B, miltefosine and paromomycin, these new and safer chemotherapy alternatives remain out of reach for the affected rural population who are most in need [5]. Moreover, the use of poor-quality drugs can be life-threatening for vulnerable patients and also have a devastating impact on public health and elimination programmes targeting the disease [6].

The glycolytic enzyme glyceraldehyde 3-phosphate dehydrogenase (GAPDH) has been considered as a target for the inhibition of protozoan parasites [7,8]. GAPDH from the pathogenic trypanosomatids *Trypanosoma brucei*, *Trypanosoma cruzi* and *Leishmania mexicana* are quite similar to each other, but have sufficient structural differences, when compared to the human enzyme, making possible the structure-based design of compounds that selectively inhibit all three trypanosomatid enzymes, but not the human homologue [7].

**Figure 1:**
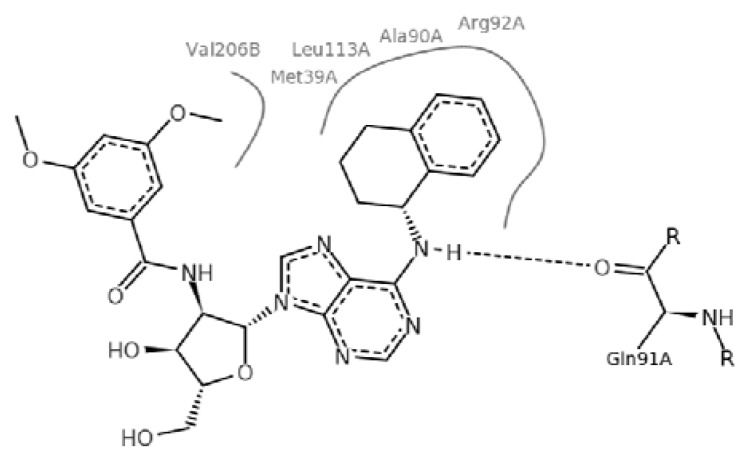
Interactions between key aminoacid residues of *Lm*GAPDH and inhibitor TND (image generated with PoseView [10], from crystallographic coordinates extracted from Protein Data Bank, code: 1I33).

By exploiting the differences in the structure of the parasitic and human GAPDH, adenosine analogs with substitutions on N-6 of the adenine ring and on the 2′ position of the ribose moiety were designed, synthesized and tested for inhibition of trypanosomatid GAPDHs, and two crystal structures of *L. mexicana* GAPDH (*Lm*GAPDH) complexed with high-affinity inhibitors that also block parasite growth were solved [9]. Induced fit of the *Lm*GAPDH backbone upon binding of the inhibitor may enlarge a cavity at the binding site to accommodate the inhibitor. The extensive hydrophobic interactions between the protein and the two substituents on the adenine scaffold of the inhibitor TND (*N*-1,2,3,4-tetrahydronaphth-1-yl-2′-[3,5-dimethoxybenzamido]-2′-deoxyadenosine), as shown in Figure 1, provide a plausible explanation for the high affinity of these inhibitors for trypanosomatid GAPDHs [9].

In order to enhance the knowledge on structural requirements for the adenosine binding to *Lm*GAPDH, structure-activity relationship studies were carried out employing different molecular modeling techniques [11,12]. In this work we have performed the calculation of a large amount of electronic, geometrical and topological descriptors with the aim to select the most relevant ones to the biological activity of adenosine compounds as inhibitors of *Lm*GAPDH, employing the recently developed variable selection algorithm OPS (Ordered Predictor Selection) [13]. By employing this strategy in conjunction with a protocol described previously [14,15], we have been able to construct a predictive model of the quantitative structure-activity relationships for the inhibition of *Lm*GAPDH by adenosine compounds.

## 2. Results and Discussion

### 2.1. Statistical Results

The OPS variable selection algorithm selected nine descriptors as the most relevant for the analysis: volume, E_HOMO_, HATS4e, HATS3u, H7m, Mor23v, BELp1, JGI2, E1v (see Table 1 for the meanings of each descriptor).

**Table 1:** Symbols, types and definitions of the selected descriptors.

Descriptor	Type	Definition
Volume	Geometric	Solvent-accessible surface-bounded molecular volume
E_HOMO_	Electronic	Energy of the highest occupied molecular orbital
HATS4e	GETAWAY	Leverage-weighted autocorrelation of lag 4/weighted by atomic Sanderson electronegativities
HATS3u	GETAWAY	Leverage-weighted autocorrelation of lag 3/unweighted
H7m	GETAWAY	H autocorrelation of lag 2/weighted by atomic masses
Mor23v	3D-MoRSE	3D-MoRSE-signal 23/weighted by atomic van der Waals volumes
BELp1	BCUT	Lowest eigenvalue n.1 of Burden matrix/weighted by atomic polarizabilities
JGI2	Galvez topological charge indices	Mean topological charge index of order 2
E1v	WHIM	1st component accessibility directional WHIM index, weighted by atomic van der Waals volumes

The PLS regression models obtained with these descriptors have resulted in the statistical parameters presented in Table 2. In order to reassure the suitability of the selected descriptors for building QSAR models for the compounds under study, other two techniques were also employed: Principal Component Regression (PCR) and Multiple Linear Regression (MLR). Statistical results for these techniques are also displayed in Table 2. There, it is possible to observe that the optimum number of latent variables for PLS is 1, while the optimal number of principal components for PCR is 2, since those are the ones presenting lowest SEV (standard error of validation) and PRESS (cross-validation predicted residual error sum of squares) values. 

Then, applying leave-one-out (LOO) cross-validation, the best PLS model presents correlation coefficients of 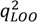 = 0.852 and ***r^2^*** = 0.874, whereas in the best PCR models these values are 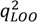 = 0.873 and ***r^2^*** = 0.852, indicating good internal consistency for both models. Leave-N-out (LNO) cross-validation results show that the models continue to present significant correlation coefficients (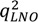 = 0.850 and 0.854 for PLS and PCR, respectively) even when 30% of the samples are left out for prediction, which indicates that robust models were obtained.

**Table 2:** Statistical parameters for the PLS, PCR and MLR models based on the 9 selected descriptors.

*PLS models*						*PCR models*					
Factors	SEV	PRESS	***r^2^***	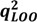	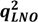 *	PCs	SEV	PRESS	***r^2^***	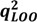	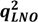 *
**1**	**0.389**	**7.105**	**0.874**	**0.852**	**0.850**	**1**	0.389	7.112	0.869	0.852	
**2**	0.401	7.571	0.885	0.843		**2**	**0.388**	**7.092**	**0.873**	**0.852**	**0.854**
**3**	0.409	7.877	0.891	0.837		**3**	0.396	7.364	0.873	0.847	
**4**	0.402	7.580	0.897	0.843		**4**	0.407	7.804	0.877	0.838	
**5**	0.402	7.599	0.899	0.843		**5**	0.402	7.602	0.883	0.842	
**6**	0.398	7.450	0.899	0.845		**6**	0.409	7.881	0.883	0.837	
**7**	0.398	7.431	0.899	0.846		**7**	0.418	8.231	0.884	0.829	
**8**	0.397	7.421	0.899	0.846		**8**	0.443	9.234	0.884	0.810	
**9**	0.397	7.416	0.899	0.846		**9**	0.397	7.416	0.899	0.846	
***MLR model***											
		***r^2^***	0.899	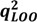	0.845	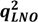 *	0.842	**RMSE**	0.397		

* Average value of N ranging from 2 to 14.

### 2.2. External Model Validation and Y-Randomization Tests

**Figure 2:**
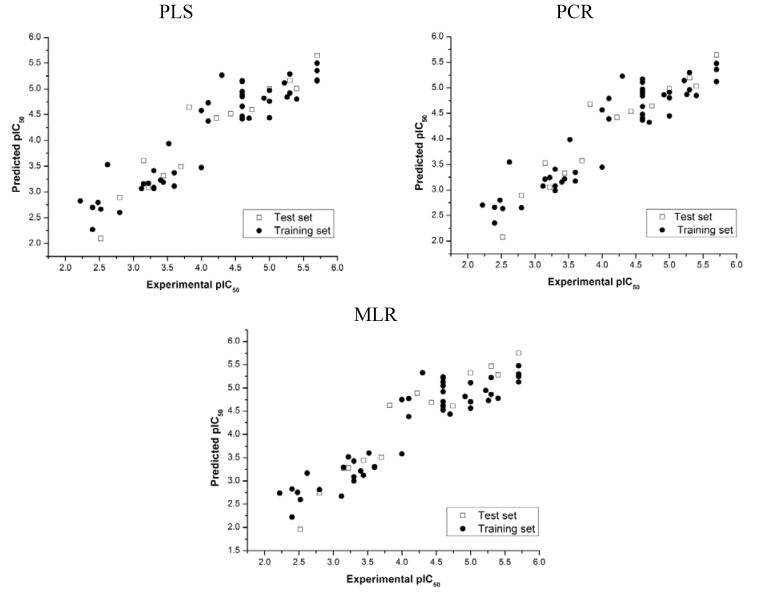
Experimental *versus* predicted pIC_50_ values of the training and test set compounds.

External validation tests were applied in order to evaluate the predictive power of the QSAR models constructed. A plot of experimental versus predicted pIC_50_ values comparing the compounds in both training and test sets, using the three regression techniques employed here, is shown in Figure 2. The good agreement between the experimental and calculated values indicates that predictive models were obtained, since good values of external validation correlation coefficients (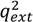) and standard errors of prediction (SEP) were achieved (see Table 3). These results indicate that the QSAR models constructed can be used to accurately predict the biological activity of other compounds within this structural class.

Chance correlations between the dependent variable and the selected descriptors were verified employing the y-randomization validation. In this test, the pIC_50_ values are scrambled and the *r^2^* and *q^2^* values are calculated. If low values for both parameters are found, then one can be sure that a true correlation of the descriptors with the response variable exists in the data set [16,17]. In the 20 y-randomizations performed for our data, only low values of *r^2^* and *q^2^* were obtained (see Table 3). So, this indicates that the descriptors selected by the OPS algorithm possess a true correlation with the dependent variable, attesting that our statistical results are not a chance correlation result.

**Table 3:** Statistical parameters of external validation and y-randomization tests.

Model	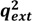	SEP	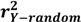 *	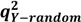 *
**PLS**	0.900	0.317	0.097	0.155
**PCR**	0.904	0.312	0.143	0.055
**MLR**	0.875	0.346	0.236	0.248

* Average value of 20 Y-randomizations.

The models obtained were ranked according to the methodology proposed by Karoly *et al.* [18,19], where ranks are compared with random numbers. The sum of ranking differences (SRD) arranges the models in such a way that low values of SRD are related to better models, while similar SRD values indicates the similarity of the models. Furthermore, the discrete distribution for a small number of objects (n < 14) is calculated, whereas the normal distribution is used as a reasonable approximation if the number of objects is large. This theoretical distribution is visualized for random numbers and can be used to identify SRD values for models that are far from being random, a procedure named as Comparison of Ranks by Random Numbers (CRNN).

**Figure 3:**
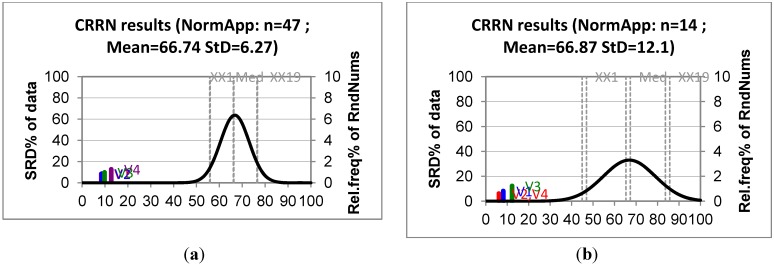
SRD-CRRN test results for (**a**) training and (**b**) test sets.

The results for the ranking procedure are presented in Table 4 for training and test sets, while Figure 3 shows the SRD distributions (data matrices are provided as supplemental material S2). These results indicate that for both training and test sets the models obtained are better (or similarly) ranked than the experimental values, and that the SRD values for models are not random.

**Table 4:** SRD ranking of models and experimental values, p% interval and percentiles output for training and test sets.

Training set				Test set			
Ranking results		p%		Ranking results		p%	
Name	SRD	x < SRD > = x		Name	SRD	x < SRD > =x	
V1 *	92	1.05 10^−18^	1.48 10^−18^	V2	6	1.19 10^−5^	3.08 10^−5^
V2	94	1.48 10^−18^	1.91 10^−18^	V4	6	1.19 10^−5^	3.08 10^−5^
V3	108	9.18 10^−18^	1.10 10^−17^	V1	8	3.08 10^−5^	7.45 10^−5^
V4	140	5.75 10^−16^	7.00 10^−16^	V3	12	1.73 10^−4^	3.88 10^−4^
XX1	618	4.80	5.06	XX1	46	4.61	5.47
Q1	684	24.67	25.64	Q1	58	24.45	27.12
Med	732	49.24	50.40	Med	66	48.78	52.08
Q3	778	74.96	75.88	Q3	74	73.59	76.22
XX19	846	94.79	95.10	XX19	84	94.77	95.59

* (V1 = PLS model, V2 = PCR model, V3 = MLR model, and V4 = experimental values).

### 2.3. Applicability Domain

The applicability domain was defined here in terms of leverage and Studentized residuals for all samples in the training set. Leverage (*h*) is a quantity that represents a sample’s distance to the centroid of the training set. For the *i_th_* sample, 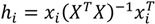 (*i =* 1,*…*, *m*), where *x_i_* is the descriptor row-vector for compound *i*, *m* is the number of query compounds, *X* is the *n* × *k* training set matrix, *k* is the number of model descriptors and *n* is the number of samples in the training set. A leverage value greater than a certain critical value for a training set sample, defined here on the basis of 95% confidence level, means that the sample has a high influence in the model.

Concerning *Y* outliers, the simple examination of raw *y* residuals can be misleading due to the effect of leverage. A sample with an extreme *y* value pulls the model towards itself, decreasing the difference between its experimental and fitted *y* values. In contrast, a sample with a *y* value lying close to the *y* mean value, having little leverage, do not greatly influence the model so its *y* residual tends to be higher. In order to have a more realistic picture, the Studentized residual, *r_i_*, can be applied, since it takes leverage into account. *r_i_* is derived from the root mean squared *y* residual for the training set (*RMSE*), and is given by Equation (1):

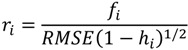
(1)


**Figure 4:**
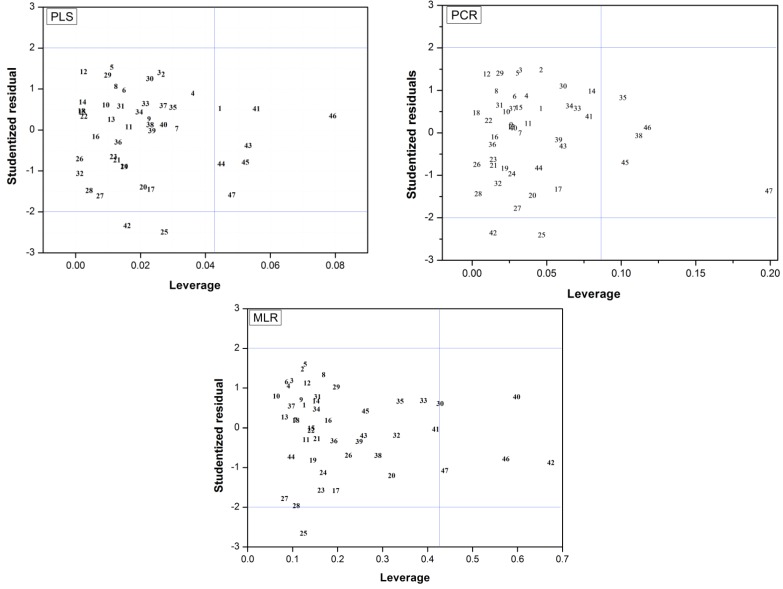
Plots of leverage *versus* Studentized residuals for the regression models constructed. Blue lines indicate the thresholds representing a probability level of 95%.

Since it is assumed that *r_i_* is normally distributed, a t test can determine whether a sample’s Studentized residual is large enough to classify such sample as a *Y* outlier. Here, a critical value for *r_i_* was computed at a 95% probability level, based on the *n* training set samples. Finally, the plots of *h_i_ versus r_i_* for the best PLS, PCR and MLR models were examined in order to determine the applicability domain of these models. Results are shown in Figure 4, where is possible to verify that none of the compounds from the training set can be considered as a response outlier, since all of them present low combined values of *h_i_* and *r_i_*. Although compound **25**, in all models, and compound **42** in PLS and PCR present high Y residuals, both of them have extremely low leverage values, meaning that this outcome does not significantly influence the model. Meanwhile, compounds with relatively high leverage values (**1**, **41**, **43**–**47** in PLS; **35**, **38**, **45**–**47** in PCR; and **40**, **42**, **46** and **47** in MLR) are inside the applicability domains of their respective models, since they are within the thresholds of *r_i_*.

### 2.4. Molecular Implications for Ligand Design

**Figure 5:**
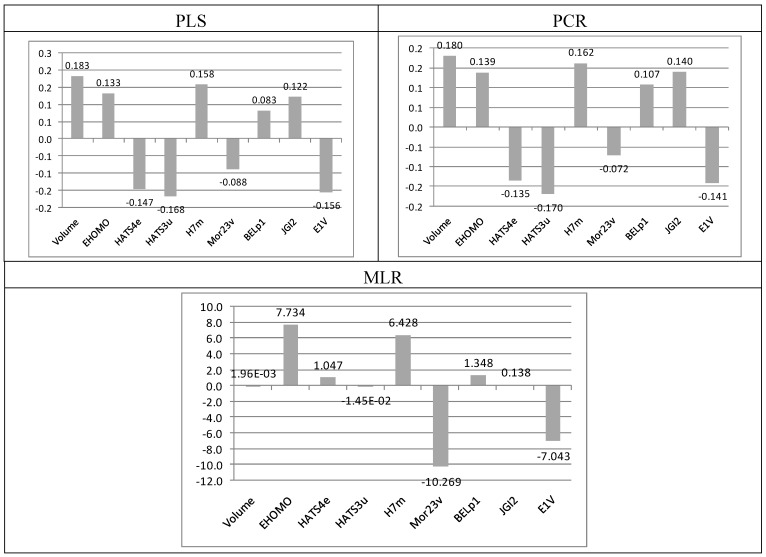
Contribution of each descriptor to the regression vector.

Since reliable QSAR models were obtained, the regression vectors can be used to analyze the selected molecular features and to suggest structural modifications that can be able to improve the biological activity of molecules similar to the ones studied here. The contributions of each descriptor to the regression vector for the best models obtained are displayed in Figure 5.

The positive regression coefficient of descriptors such as Volume, E_HOMO_, H7m, BELp1, and JGI2 indicates that their higher values are favorable for the *Lm*GAPDH inhibition. Then, a given molecule must present a high solvent-accessible surface-bounded molecular volume (as defined by Connoly [20]) in order to show affinity to *Lm*GAPDH. Molecular volume is an useful index for understanding the drug action since short range dispersion forces play a major role in the binding of ligands to biological receptors. For efficient and specific binding, the receptor cavity must be filled with the interacting ligand in the most optimal geometry [21]. Additionally, in this case, E_HOMO_ must also have a high value, which indicates that a highly active molecule must be the one with a high ionization potential, meaning that it would easily donate an electron in a charge transfer mechanism [22].

Geometry, Topology and Atom-Weight Assembly (GETAWAY) descriptors such as H7m try to match 3D-molecular geometry provided by the molecular influence matrix and molecular topology with chemical information by using different atomic weightings (atomic mass, polarizability, van der Waals volume, and electronegativity) [23]. The information provided by the H7m descriptor in our PLS model is weighted by atomic masses, having a positive influence on the biological activity.

BELp1 is also a 2D descriptor from the class of BCUT descriptors, which accounts for the first eight lowest absolute eigenvalues for the modified Burden adjacency matrix, where p refers to atomic polarizability and 1 is the eigenvalue rank. The ordered sequence of the lowest eigenvalues reflects the relevant aspects of molecular structure, which are useful for similarity searching [24]. JGI2 belongs to GALVEZ descriptors, which are the Galvez topological charge indices, and have their origin in the first 10 eigenvalues of the polynomial of corrected adjacency matrix of the compounds. JGI2 represents the mean topological charge index of order 2 [25].

On the other hand, from the negative signs of regression coefficients of HATS4e, HATS3u, Mor23v and E1v, it is evident that these descriptors contribute negatively to the biological activity of adenosine compounds. Thus, lower values of these descriptors are required in order to obtain high activity compounds. HATS4e and HATS3u also belong to the class of GETAWAY descriptors. The HATS prefix means leverage-weighted autocorrelation, 4 and 3 are the lag numbers, and while HATS4e is weighted by atomic Sanderson electronegativities, HATS3u is unweighted [26]. The selection of the 3D descriptors Mor23v can be related to the importance of molecular conformation of adenosine analogues for the interaction with key amino acids from the binding site of GAPDH [27]. E1v belongs to the class of Weighted Holistic Invariant Molecular (WHIM) descriptors, which contain 3D information calculated from the x,y,z-coordinates. E1v is the 1st component accessibility directional WHIM index, weighted by atomic van der Waals volumes [28]. 

On the basis of the foregoing considerations, it is possible to observe a balance between steric and electrostatic properties influencing the affinity of adenosines to *Lm*GAPDH, which is in agreement with the findings of Guido *et al.* [11]. Steric molecular features are represented by volume, H7m, E1v, and Mor23v, while descriptors E_HOMO_, HATS4e, BELp1, and JGI2 account for electronic aspects.

## 3. Experimental

### 3.1. Data Sets

The 61 adenosine derivatives employed in this study were selected from the literature [7,29,30,31,32]. IC_50_ values, measured under the same experimental conditions, were converted to the corresponding pIC_50_ (-logIC_50_), and used as dependent variable in the regression analyses. Structures and pIC_50_ values for all compounds are displayed in Table 5. From the whole data set, 47 compounds were selected to constitute the training set, while 14 compounds were taken to compose a test set to be utilized in an external validation procedure. This selection was performed carefully in order to certify that the structural diversity and the pIC_50_ distribution of the data set were well represented in both training and test sets.

**Table 5:** Chemical structures and pIC_50_ values for training and test set compounds.

Training set compounds					
Cpd	Structure	pIC_50_	Cpd	Structure	pIC_50_
**1**	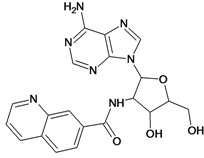	3.30	**2**	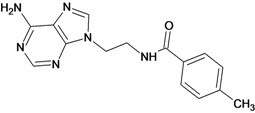	2.40
**3**	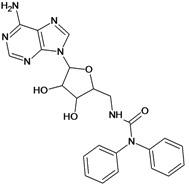	3.60	**4**	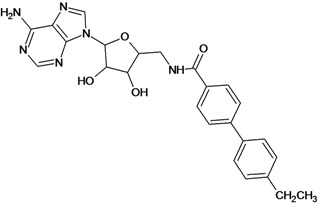	3.12
**5**	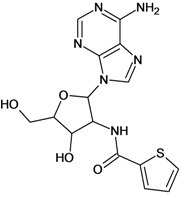	2.62	**6**	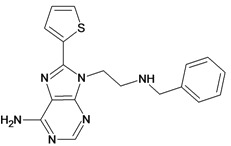	3.40
**7**	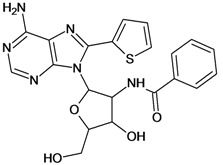	3.30	**8**	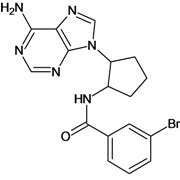	3.15
**9**	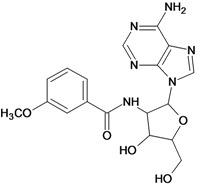	3.52	**10**	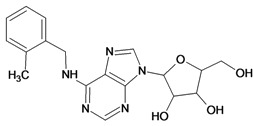	3.15
**11**	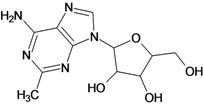	2.22	**12**	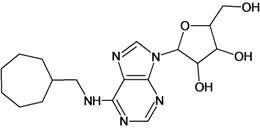	2.74
**13**	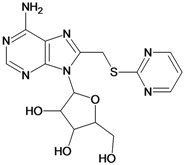	2.40	**14**	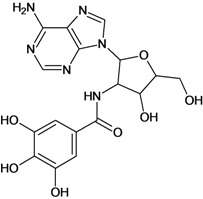	3.60
**15**	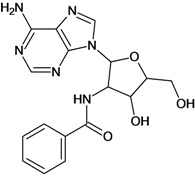	2.48	**16**	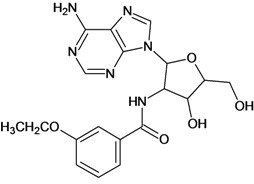	3.40
**17**	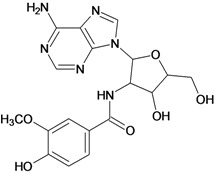	3.30	**18**	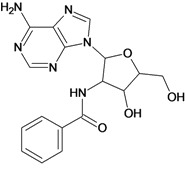	2.52
**19**	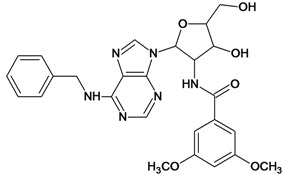	4.70	**20**	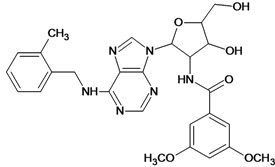	4.60
**21**	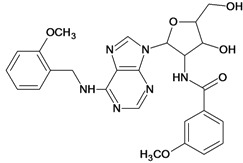	4.60	**22**	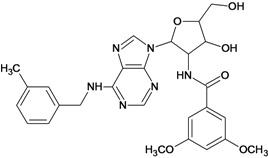	4.60
**23**	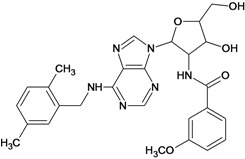	4.60	**24**	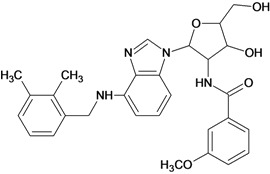	5.26
**25**	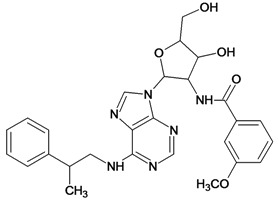	4.10	**26**	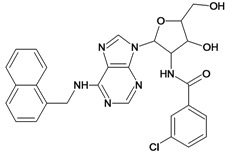	5.00
**27**	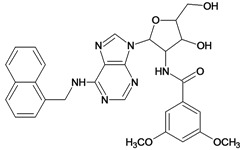	5.70	**28**	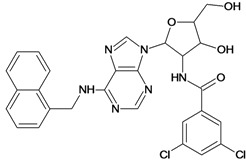	4.92
**29**	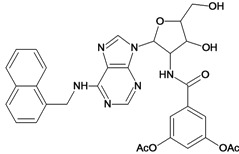	5.00	**30**	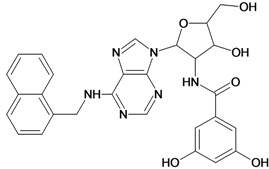	5.30
**31**	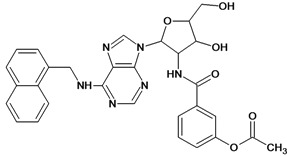	4.30	**32**	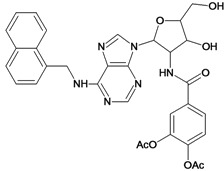	4.60
**33**	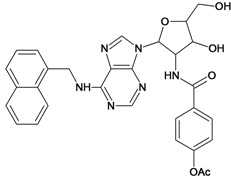	4.00	**34**	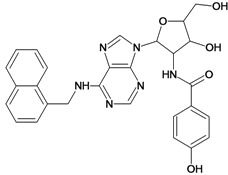	4.10
**35**	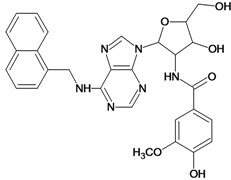	5.00	**36**	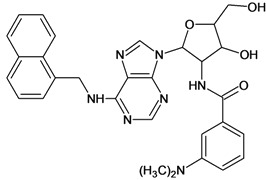	4.60
**37**	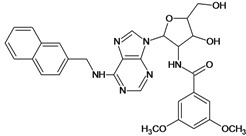	5.70	**38**	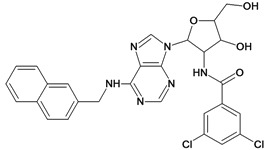	4.60
**39**	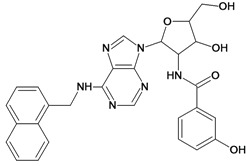	4.60	**40**	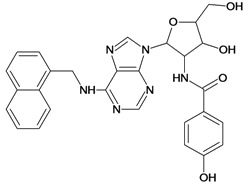	4.00
**41**	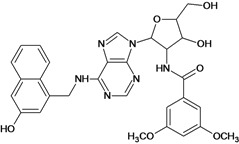	5.70	**42**	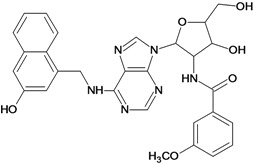	5.22
**43**	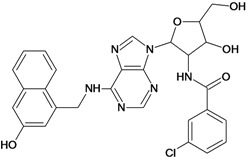	5.40	**44**	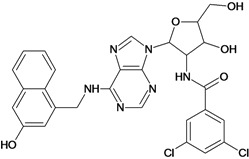	4.60
**45**	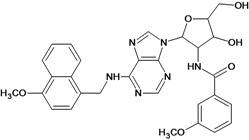	5.30	**46**	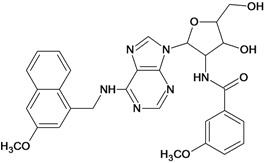	5.70
**47**	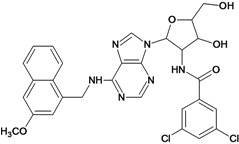	4.60	**48**	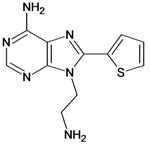	2.80
**49**	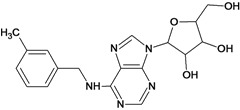	3.15	**50**	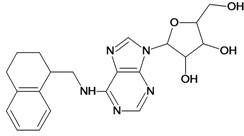	3.44
**51**	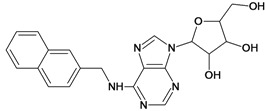	3.82	**52**	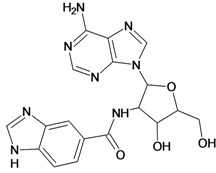	2.52
**53**	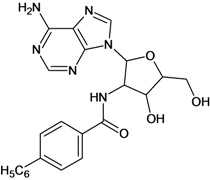	3.70	**54**	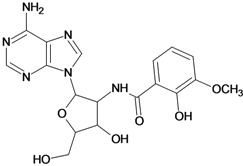	3.22
**55**	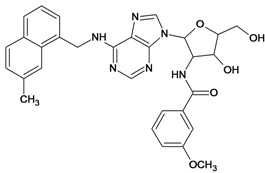	5.30	**56**	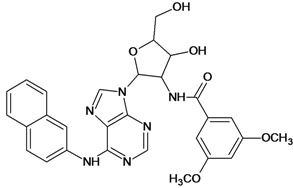	4.22
**57**	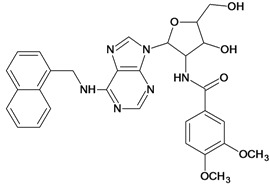	5.40	**58**	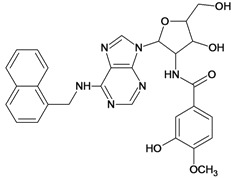	4.43
**59**	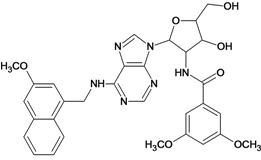	5.70	**60**	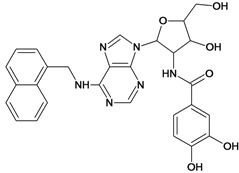	4.74
**61**	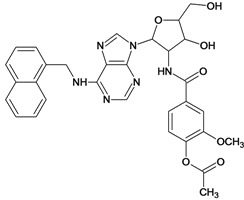	5.00			

### 3.2. Descriptor Calculation and Selection

A pre-optimization of the geometries of all compounds were carried out with the semiempirical method PM3 [33,34]. A final optimization was performed with the density functional theory (DFT) using the B3LYP functional [35,36] along with the 6-311G** basis sets [37]. Several electronic descriptors were calculated using Gaussian 03 [37], and various structural descriptors were calculated with the QSAR module implemented in HyperChem 4.5 [34]. A set of 1,100 molecular descriptors, encoding information about molecular structure, connectivity and topology were also calculated with Dragon 5.4 [38]. All descriptors were autoscaled in order to give them the same weight in the analyses.

With the aim to reduce the number of descriptors, the absolute values of correlation coefficients between each descriptor and pIC_50_ were calculated. Descriptors with coefficients lower than 0.3 were eliminated from the analysis, and so 72 descriptors remained. From this subset of descriptors, the ones presenting a non-uniform distribution related to the pIC_50_ were also eliminated, leaving 35 descriptors in the analysis. Then, the Ordered Predictor Selection (OPS) algorithm [13] was employed to perform a variable selection. The basic idea of this algorithm is to attribute an importance to each descriptor based on an informative vector. The columns of the matrix are rearranged in such a way that the most important descriptors are presented in the first columns. Afterwards, successive PLS regressions are performed with an increasing number of descriptors in order to find the best PLS model. In this analysis, the regression vector was used as an informative vector and the correlation coefficient of cross-validation, *q^2^*, as a criterion to select the best models. The suitability of the descriptors selected by this procedure was tested by performing Principal Component Regression (PCR) and Multiple Linear Regression (MLR).

The best models were chosen on the basis of the cross-validation predicted residual error sum of squares (PRESS), being the optimal number of PLS or PCR components the one that minimizes PRESS. Model quality was verified mainly by the correlation coefficients *r^2^* and *q^2^* and also by the prediction residuals, which are indications that the model can be used for making predictions of the biological properties of unknown compounds, which are structurally similar. Model robustness and sensitiveness were additionally evaluated by applying leave-N-out (LNO) cross-validation and y-randomization tests. It is important to mention that the model validation is a very crucial step in QSAR studies [39,40,41,42]. In the LNO cross-validation procedure, N compounds (N varying from 1 to 20) were left out from the training set. For a particular N, the data were randomized 30 times, and the average and standard deviation values for *q^2^* were used. In the y-randomization, the dependent variable-vector was scrambled 20 times in order to verify the occurrence of chance correlations between the dependent variable and the selected descriptors [16,17]. Applicability domain was defined through the examination of the plots of leverage *versus* Studentized residuals for the best PLS, PCR and MLR models.

## 4. Conclusions

The continuous search for new antileishmanial compounds is undoubtedly important for the researches in neglected diseases. In this context, QSAR models can play an important role in the discovery and optimization of new drug candidates. In this work, PLS, PCR and MLR models were developed to provide indications on relevant molecular features for the antileishmanial activity of adenosine compounds. A set of nine descriptors selected by the OPS approach have demonstrated to be suitable for the construction of QSAR models. The models constructed can be used by researchers interested in synthesizing new adenosine compounds. Once a new adenosine compound is designed, its structure can be submitted to the calculations performed in our work, *i.e.*, the variables selected in our study can be calculated for this new compound. Then, the values of these variables can be inserted into the regression models in order to predict the pIC_50_ for this compound. So, our models can be helpful to decide which compounds should be synthesized, saving time and resources. The good statistical parameters, stability and robustness of the models obtained, as assured by the validation tests applied over our data, indicate that these models can be used to design other adenosine derivatives with improved antileishmanial activity.

## Supplementary Materials

Supplementary materials can be accessed at: http://www.mdpi.com/1420-3049/18/5/5032/s1.
