# Supplementary material for: Identification of Electronic and Structural Descriptors of Adenosine Analogues Related to Inhibition of Leishmanial Glyceraldehyde-3-Phosphate Dehydrogenase
Source: Molecules. 2013 Apr 29;18(5):5032–50. doi: 10.3390/molecules18055032 (PMC6269754; doi:10.3390/molecules18055032)
Supplement: Supplementary file 1 [file molecules-18-05032-s001.pdf]

Supplemental Materials

Table S1. Experimental and predicted pIC_{50} values.

Training set compounds				
**Compound**	**Experimental pIC_{50}**	**Predicted pIC_{50} (PLS)**	**Predicted pIC_{50} (PCR)**	**Predicted pIC_{50} (MLR)**
**1**	5.70	5.50	5.47	5.48
**2**	5.70	5.17	5.12	5.13
**3**	5.70	5.15	5.12	5.25
**4**	5.70	5.35	5.36	5.30
**5**	5.40	4.80	4.85	4.78
**6**	5.30	4.92	4.96	4.86
**7**	5.30	5.29	5.30	5.23
**8**	5.26	4.84	4.87	4.73
**9**	5.22	5.11	5.14	4.95
**10**	5.00	4.76	4.80	4.70
**11**	5.00	4.97	4.91	5.11
**12**	5.00	4.44	4.45	4.56
**13**	4.92	4.82	4.86	4.82
**14**	4.70	4.43	4.32	4.43
**15**	4.60	4.43	4.37	4.60
**16**	4.60	4.66	4.63	4.53
**17**	4.60	5.16	5.11	5.24
**18**	4.60	4.42	4.41	4.53
**19**	4.60	4.95	4.93	4.92
**20**	4.60	5.14	5.17	5.13
**21**	4.60	4.88	4.90	4.71
**22**	4.60	4.47	4.48	4.62
**23**	4.60	4.85	4.84	5.22
**24**	4.60	4.94	4.97	5.05
**25**	4.30	5.26	5.23	5.33
**26**	4.10	4.37	4.39	4.38
**27**	4.10	4.73	4.79	4.77
**28**	4.00	4.58	4.56	4.75
**29**	4.00	3.48	3.45	3.58
**30**	3.60	3.11	3.17	3.31
**31**	3.60	3.37	3.34	3.29
**32**	3.52	3.94	3.99	3.60
**33**	3.44	3.19	3.22	3.12
**34**	3.40	3.23	3.15	3.22
**35**	3.30	3.09	2.99	3.00
**36**	3.30	3.41	3.40	3.43
**37**	3.30	3.07	3.07	3.09
**38**	3.22	3.17	3.24	3.52
**39**	3.15	3.16	3.21	3.29

Table S1. *Cont.*

Compound	Experimental pIC_{50}	Predicted pIC_{50} (PLS)	Predicted pIC_{50} (PCR)	Predicted pIC_{50} (MLR)
**Test set compounds**				
**40**	3.12	3.07	3.07	2.67
**41**	2.80	2.60	2.65	2.81
**42**	2.62	3.53	3.55	3.17
**43**	2.52	2.66	2.64	2.60
**44**	2.48	2.80	2.80	2.76
**45**	2.40	2.70	2.66	2.22
**46**	2.40	2.27	2.35	2.83
**47**	2.22	2.83	2.70	2.74
**48**	5.70	5.65	5.64	5.75
**49**	5.40	5.01	5.03	5.28
**50**	5.30	5.16	5.19	5.47
**51**	5.00	5.00	4.99	5.32
**52**	4.74	4.60	4.64	4.61
**53**	4.43	4.52	4.54	4.68
**54**	4.22	4.43	4.42	4.89
**55**	3.82	4.65	4.68	4.62
**56**	3.70	3.49	3.57	3.51
**57**	3.44	3.31	3.33	3.45
**58**	3.22	3.09	3.05	3.28
**59**	3.15	3.61	3.53	3.28
**60**	2.80	2.89	2.89	2.75
**61**	2.52	2.10	2.08	1.96

Table S2. Calculation of ranks, absolute values of differences and their sums.

					Training set									
	**Average**	**rnk**	**V1**	**rnk1**	**diff1**	**V2**	**rnk2**	**diff2**	**V3**	**rnk3**	**diff3**	**V4**	**rnk4**	**diff4**
46	2.46	1	2.26866	1	0	2.351	1	0	2.83	7	6	2.4	2	1
45	2.50	2	2.69998	4	2	2.66	4	2	2.222	1	1	2.4	3	1
43	2.60	3	2.66338	3	0	2.637	2	1	2.599	2	1	2.52	5	2
47	2.62	4	2.8263	6	2	2.704	5	1	2.737	4	0	2.22	1	3
44	2.71	5	2.79536	5	0	2.798	6	1	2.755	5	0	2.48	4	1
41	2.72	6	2.59998	2	4	2.651	3	3	2.814	6	0	2.8	7	1
40	2.98	7	3.06781	7	0	3.073	8	1	2.674	3	4	3.12	8	1
35	3.09	8	3.08546	9	1	2.986	7	1	3.001	8	0	3.3	11	3
37	3.13	9	3.06854	8	1	3.074	9	0	3.091	9	0	3.3	12	3
39	3.20	10	3.15542	11	1	3.21	12	2	3.292	14	4	3.15	9	1
42	3.22	11	3.52814	18	7	3.546	18	7	3.171	11	0	2.62	6	5
33	3.24	12	3.18878	13	1	3.216	13	1	3.122	10	2	3.44	15	3
34	3.25	13	3.2277	14	1	3.155	10	3	3.215	12	1	3.4	14	1
38	3.29	14	3.16862	12	2	3.243	14	0	3.518	17	3	3.22	10	4
30	3.30	15	3.11208	10	5	3.174	11	4	3.308	15	0	3.6	17	2
36	3.39	16	3.41321	16	0	3.404	16	0	3.429	16	0	3.3	13	3
31	3.40	17	3.37056	15	2	3.341	15	2	3.291	13	4	3.6	18	1
29	3.63	18	3.47506	17	1	3.445	17	1	3.583	18	0	4	19	1
32	3.76	19	3.93572	19	0	3.986	19	0	3.601	19	0	3.52	16	3
26	4.31	20	4.37487	20	0	4.392	22	2	4.381	20	0	4.1	21	1
14	4.47	21	4.43016	23	2	4.324	20	1	4.434	21	0	4.7	34	13
28	4.47	22	4.57751	26	4	4.565	26	4	4.75	30	8	4	20	2
18	4.49	23	4.41525	21	2	4.409	23	0	4.527	23	0	4.6	24	1
15	4.50	24	4.42738	22	2	4.365	21	3	4.602	25	1	4.6	25	1
22	4.54	25	4.46704	25	0	4.482	25	0	4.622	26	1	4.6	26	1
27	4.60	26	4.72796	28	2	4.791	28	2	4.77	31	5	4.1	22	4
16	4.60	27	4.66037	27	0	4.634	27	0	4.525	22	5	4.6	27	0
12	4.61	28	4.43973	24	4	4.449	24	4	4.561	24	4	5	36	8
21	4.77	29	4.88470	34	5	4.899	34	5	4.705	28	1	4.6	28	1
10	4.82	30	4.76018	29	1	4.802	29	1	4.702	27	3	5	37	7
19	4.85	31	4.94794	37	6	4.925	36	5	4.917	35	4	4.6	29	2
13	4.85	32	4.8172	31	1	4.862	32	0	4.817	33	1	4.92	35	3
23	4.88	33	4.85248	33	0	4.844	30	3	5.219	41	8	4.6	30	3
24	4.89	34	4.94434	36	2	4.975	38	4	5.045	37	3	4.6	31	3
8	4.92	35	4.8423	32	3	4.869	33	2	4.728	29	6	5.26	40	5
5	4.96	36	4.80034	30	6	4.847	31	5	4.777	32	4	5.4	43	7
11	5.00	37	4.96811	38	1	4.911	35	2	5.112	38	1	5	38	1
20	5.01	38	5.14051	40	2	5.17	43	5	5.126	39	1	4.6	32	6
6	5.01	39	4.91854	35	4	4.961	37	2	4.86	34	5	5.3	41	2
17	5.03	40	5.16119	42	2	5.109	39	1	5.236	43	3	4.6	33	7
25	5.03	41	5.26368	44	3	5.229	44	3	5.325	46	5	4.3	23	18
9	5.11	42	5.11402	39	3	5.142	42	0	4.947	36	6	5.22	39	3
7	5.28	43	5.28582	45	2	5.299	45	2	5.225	42	1	5.3	42	1

Table S2. *Cont.*

						**Training set**							
	**Average**	**rnk**	**V1**	**rnk1**	**diff1**	**V2**	**rnk2**	**diff2**	**V3**	**rnk3**	**diff3**	**V4**	**rnk4**	**diff4**
2	5.28	44	5.17056	43	1	5.121	41	3	5.128	40	4	5.7	44	0
3	5.31	45	5.15466	41	4	5.12	40	5	5.247	44	1	5.7	45	0
4	5.43	46	5.35338	46	0	5.359	46	0	5.298	45	1	5.7	46	0
1	5.54	47	5.49568	47	0	5.475	47	0	5.477	47	0	5.7	47	0
				Sum	92			94			108			140
61	2.16	1	2.09768	1	0	2.075	1	0	1.959	1	0	2.52	1	0
60	2.83	2	2.89118	2	0	2.894	2	0	2.745	2	0	2.8	2	0
58	3.16	3	3.08913	3	0	3.048	3	0	3.279	4	1	3.22	4	1
57	3.38	4	3.31273	4	0	3.328	4	0	3.446	5	1	3.44	5	1
59	3.39	5	3.60661	6	1	3.527	5	0	3.277	3	2	3.15	3	2
56	3.57	6	3.49328	5	1	3.571	6	0	3.509	6	0	3.7	6	0
55	4.44	7	4.64639	10	3	4.684	10	3	4.624	8	1	3.82	7	0
54	4.49	8	4.43297	7	1	4.425	7	1	4.887	10	2	4.22	8	0
53	4.54	9	4.5185	8	1	4.538	8	1	4.683	9	0	4.43	9	0
52	4.65	10	4.59673	9	1	4.645	9	1	4.613	7	3	4.74	10	0
51	5.08	11	5.00052	11	0	4.985	11	0	5.321	12	1	5	11	0
49	5.18	12	5.00585	12	0	5.034	12	0	5.281	11	1	5.4	13	1
50	5.28	13	5.16101	13	0	5.194	13	0	5.469	13	0	5.3	12	1
48	5.68	14	5.64711	14	0	5.644	14	0	5.746	14	0	5.7	14	0
				Sum	8			6			12			6
